# microRNA-dependent regulation of gene expression in GABAergic interneurons

**DOI:** 10.3389/fncel.2023.1188574

**Published:** 2023-05-05

**Authors:** Karolina Anna Kołosowska, Gerhard Schratt, Jochen Winterer

**Affiliations:** ^1^Department of Neurochemistry, Institute of Psychiatry and Neurology, Warsaw, Poland; ^2^Lab of Systems Neuroscience, Department of Health Science and Technology, Institute for Neuroscience, Swiss Federal Institute of Technology ETH, Zurich, Switzerland

**Keywords:** microRNA, interneurons, neural circuits, neurodevelopment, neuropsychiatric disorders

## Abstract

Information processing within neuronal circuits relies on their proper development and a balanced interplay between principal and local inhibitory interneurons within those circuits. Gamma-aminobutyric acid (GABA)ergic inhibitory interneurons are a remarkably heterogeneous population, comprising subclasses based on their morphological, electrophysiological, and molecular features, with differential connectivity and activity patterns. microRNA (miRNA)-dependent post-transcriptional control of gene expression represents an important regulatory mechanism for neuronal development and plasticity. miRNAs are a large group of small non-coding RNAs (21–24 nucleotides) acting as negative regulators of mRNA translation and stability. However, while miRNA-dependent gene regulation in principal neurons has been described heretofore in several studies, an understanding of the role of miRNAs in inhibitory interneurons is only beginning to emerge. Recent research demonstrated that miRNAs are differentially expressed in interneuron subclasses, are vitally important for migration, maturation, and survival of interneurons during embryonic development and are crucial for cognitive function and memory formation. In this review, we discuss recent progress in understanding miRNA-dependent regulation of gene expression in interneuron development and function. We aim to shed light onto mechanisms by which miRNAs in GABAergic interneurons contribute to sculpting neuronal circuits, and how their dysregulation may underlie the emergence of numerous neurodevelopmental and neuropsychiatric disorders.

## Introduction

Cortical information processing depends on intricately and dynamically interconnected neuronal circuits composed of (1) glutamatergic excitatory neurons (or principal neurons), and (2) γ-aminobutyric acid (GABA)ergic inhibitory interneurons (INs) (Wood et al., 2017; Swanson and Maffei, 2019). GABAergic INs are a highly heterogeneous neuronal population that can be further divided into distinct subtypes based on morphology, molecular markers, electrophysiological properties, and connectivity (Ascoli et al., 2008; Lim et al., 2018; Mihaljević et al., 2019). Whilst principal neurons signal within and among various brain regions, the majority of cortical GABAergic INs are considered to project mainly locally (but see descriptions of long-range projecting GABAergic INs (Jinno et al., 2007; Caputi et al., 2013). In this manner they control local network activity by gating information flow and contributing to sculpting network dynamics (Teppola et al., 2019). Examples of such functions include the maintenance of excitatory and inhibitory (E/I) balance, the generation and synchronization of cortical rhythms, as well as the modulation of cortical circuit plasticity (Tremblay et al., 2016; Fishell and Kepecs, 2020).

The generation and integration of the accurate number and IN subtype during relevant developmental time windows underlies the proper functioning of neural circuitry. A large amount of evidence indicates that the expression of particular genetic programmes confers structural and functional IN diversity, which becomes evident after IN precursors become postmitotic (Shi et al., 2021; Bugeon et al., 2022). Subsequently, during migration and final position settling, extrinsic local cues shape subtype identity of cortical INs, thereby determining morphology and corollary their connectivity patterns (Guo and Anton, 2014; Peyre et al., 2015; Mayer et al., 2018; Mi et al., 2018; Fishell and Kepecs, 2020). Moreover, it has been demonstrated, that INs can change their molecular profile based on their engagement in local circuits (Donato et al., 2013; Dehorter et al., 2015, 2017). Consequently, disturbances in IN development and mature function are reflected in their misspecification and misplacement, in alterations of their morphology and connectivity as well as in their inability to change and adapt their gene expression profile in context-specific brain activity (Volk et al., 2015; Dienel and Lewis, 2019; Mukherjee et al., 2019; Iannone and De Marco García, 2021). In line with the importance of INs for circuit function, developmental disturbances, or disruptions of mature IN function have emerged as pathophysiological substrates implicated in neurodevelopmental and neuropsychiatric disorders, such as schizophrenia, depression, epilepsy, and autism spectrum disorders (Brown et al., 2015; Nelson and Valakh, 2015; Del Pino et al., 2018; Selten et al., 2018; Juric-Sekhar and Hevner, 2019; Mukherjee et al., 2019).

Post-transcriptional mechanisms play critical roles in the control of gene expression during neuronal development and function. Compared to transcriptional regulation, post-transcriptional control of gene expression allows for faster responses to environmental cues, and in addition is not restricted to the nucleus. Recently, a group of small, non-coding RNAs, known as microRNAs (miRNAs), has been highlighted as a vital and ubiquitous layer of post-transcriptional control of gene expression. miRNAs base-pair to complementary sequences in their target mRNA molecules and inhibit their translation or promote degradation (Bartel, 2018). miRNAs are particularly abundant in the brain, where they contribute to proteomic diversity across regions and are important mediators of synaptic plasticity (Schratt et al., 2006; Schratt, 2009; Aksoy-Aksel et al., 2014; Ye et al., 2016). Numerous studies have shown their fundamental involvement at different stages of neuronal development and in the control of mature neuronal functions (Kosik, 2006; Fineberg et al., 2009; McNeill and Van Vactor, 2012; Zahr et al., 2019; Zolboot et al., 2021; Chan et al., 2022). Their expression and activity are often dysregulated in pathological states resulting in a shift of the cellular and extracellular miRNA patterns. Therefore, miRNA profiling along with the analysis of their target signaling pathways has emerged as a promising approach to study the pathogenesis of many diseases (Chen et al., 2010; Geekiyanage et al., 2012; Bencurova et al., 2017; Paul et al., 2018; Figueiredo et al., 2022; Khan and Saraya, 2022; Wei and Shetty, 2022).

Notwithstanding the considerable amount of knowledge on the role of miRNA in pyramidal neuron development and plasticity, little is known on how miRNAs govern fundamental aspects of cortical inhibition. There are a few studies pinpointing the importance of miRNA regulation in GABAergic IN development and mature functions. In this review, we assemble and arrange recent data regarding miRNA-dependent gene regulation of GABAergic IN activity, with the aim to shed light onto mechanisms by which miRNA-dependent control of gene expression in INs contributes to sculpting brain circuit dynamics. We propose that elucidating miRNA-associated signaling networks may offer a powerful platform for understanding mechanisms leading to impairments of cortical INs in neurodevelopmental and neuropsychiatric disorders, such as schizophrenia and autism (Tu et al., 2018; Lim et al., 2021).

## microRNAs as gene expression regulators

miRNAs constitute a subclass of small (approximately 19–24 nucleotides in length), single-stranded non-coding RNAs that regulate post-transcriptional gene expression by repressing translation or promoting degradation of their target mRNAs. The early 1990s discovery of the first two miRNAs, lin-4 and let-7, involved in the regulation of the nematode *Caenorhabditis elegans* development, has attracted significant interest and marked a crucial milestone in molecular neurobiology by introducing a new level for controlling gene expression (Lee et al., 1993; Wightman et al., 1993). Subsequently, a growing number of miRNAs have been successively identified through various computational and experimental methods in species ranging from plants to humans. In 2002, miRBase, a miRNA registry was launched to serve as the main online repository for information regarding all potential miRNA sequences, nomenclature, classification, and target prediction (Griffiths-Jones, 2004). The most recent release of miRBase (v22) contains 48 860 mature miRNA sequences from 271 organisms. More than 2,500 mature miRNAs have been discovered in the human genome (Kozomara et al., 2019) and the expression of up to 60% of human protein-coding genes is predicted to be modulated by miRNAs (Friedman et al., 2009; Akhtar et al., 2016).

**FIGURE 1:**
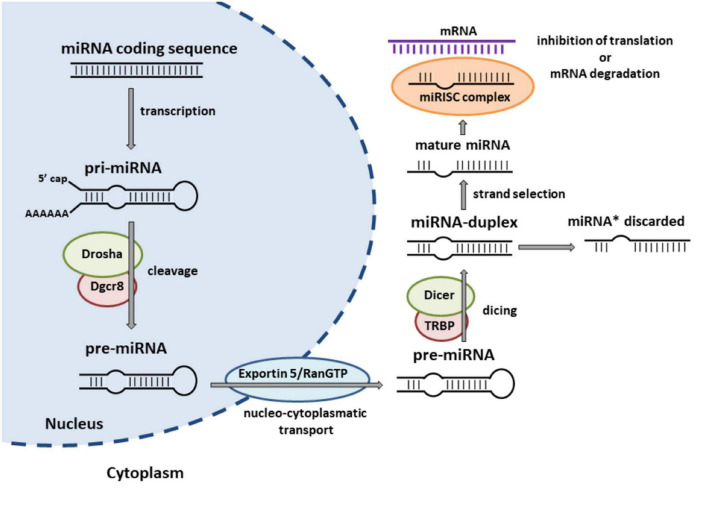
Canonical miRNA biogenesis pathway. miRNAs are initially transcribed by RNA Polymerase II/III into long primary transcripts (pri-miRNAs) that typically include a poly(A) tail and secondary hairpin structure. The pri-miRNA is subsequently cleaved by the microprocessor complex, containing the endonuclease Drosha and its partner protein Dgcr8/Pasha to the stem-loop precursor miRNA (pre-miRNA), which is then exported to the cytoplasm by exportin 5/RanGTP complex. In the cytoplasm, another RNase III enzyme, Dicer/TRBP, cuts the hairpin structure of the pre-miRNA to a miRNA duplex. One strand of the miRNA duplex is selectively incorporated into the miRNA-induced silencing complex (miRISC) and identified as the “miRNA” or “guide” strand. The other strand (originally named as “miRNA*”) is often not incorporated into a functional miRISC and subsequently degraded in the cytoplasm. Within the miRISC, miRNAs bind to complementary sequences of target mRNAs to repress their translation or induce their degradation. Adapted from Winter et al. (2009).

Most miRNAs are deployed over the genome and transcribed as individual genes, while some of them are clustered and co-expressed as polycistronic units under the control of the same promoter (Truscott et al., 2016). According to their genomic location, which determines their transcriptional regulation, miRNAs can be classified into intragenic and intergenic miRNAs (Liu et al., 2019). Intragenic miRNAs are positioned within protein-coding or non-coding genes (so called host genes) at different gene regions and are supposed to be co-transcribed with their host genes by Polymerase II (Liu et al., 2019). Conversely, intergenic miRNAs are inserted between genes and transcribed from their own Polymerase II/III promoters (Liu et al., 2019). miRNAs are first transcribed as long primary transcripts, which then undergo a series of sequential processes leading to the generation of mature miRNA (Lee et al., 2002, 2004; Bartel, 2004; Denli et al., 2004; Lund et al., 2004; Okamura et al., 2004; Okada et al., 2009; Ha and Kim, 2014; O’Brien et al., 2018; Medley et al., 2021; Ergin and Çetinkaya, 2022). For a more detailed description of the biogenesis of miRNAs see Figure 1. In addition to the canonical pathway of miRNA biogenesis, various alternative mechanisms that may omit some of the canonical steps (so called non-canonical biogenesis pathways) can produce miRNAs (Yang and Lai, 2011; Cipolla, 2014; Ha and Kim, 2014; Stavast and Erkeland, 2019) and have been shown to be involved in different human diseases, including cancer (reviewed by Liu et al., 2019).

Target specificity of the miRNA-induced silencing complex (miRISC) is determined by the sequence complementarity between the miRNA strand and the target mRNA. The sequence primarily involved in miRNA–mRNA recognition—the “seed” region—is usually composed of 6–8 nucleotides of the 5′ region of the miRNA (Chen et al., 2008; Bartel, 2009). Due to the small size of the “seed” region and the length of 3′ UTRs, miRNAs may have hundreds of mRNA targets, and any given target may be under the control of numerous miRNAs (Friedman et al., 2009). The target mRNA may be “deactivated” by one or more of the following processes: (1) endonucleolytic cleavage of the mRNA strand, (2) destabilization of the mRNA through shortening of its poly(A) tail and decapping, followed by exonucleolytic cleavage, and (3) less efficient translation into proteins on ribosomes (Fabian et al., 2010). However, endonucleolytic cleavage happens only in the rare case of perfect complementarity between miRNAs and their target mRNA. Otherwise, there is usually a combination of degradation and translational inhibition. The degree by which each of these mechanisms contributes to silencing of mRNAs is variable and not easily deduced from the geometry of the miRNA/mRNA pair.

## miRNAs in the neuron

miRNAs are present in many mammalian cell types and in various biological fluids within cells (e.g., peripheral blood mononuclear cells, PBMCs), or in the form of exosomes and as extracellular circulating miRNAs (Lagos-Quintana et al., 2002; Kriegel et al., 2013). They are highly abundant in the brain, where they significantly contribute to the functional proteomic diversity across cells and regions. miRNA interactions with their target mRNAs depend not only on sequence complementarity, but also on spatial proximity, which contributes to efficient regulation of local protein synthesis (Jansen, 2001; Martin and Ephrussi, 2009). miRNAs are highly abundant in dendrites and axons (Martin and Zukin, 2006; Kye et al., 2007; Schratt, 2009). While regulation of transcription is spatially restricted to the nucleus, miRNAs may fine-tune protein synthesis in remote subcellular compartments such as synapses (Dubes et al., 2019). The local repertoire of mRNAs preserves protein homeostasis for physiological processes and in response to intracellular and environmental cues (Das et al., 2021), and miRNA biogenesis and function themselves are subject to activity-dependent regulation (Aksoy-Aksel et al., 2014; Sambandan et al., 2017; Zampa et al., 2018). As a result, each synapse may be autonomously altered in structure and function during synaptic plasticity processes (Martin and Zukin, 2006). Furthermore, the miRNA biogenesis machinery is not restricted to the soma. For example, specific pre-miRNAs can be transported into the synapto-dendritic compartment (Bicker et al., 2013) and cleaved at the synapse to mature miRNAs (Lugli et al., 2008, Sambandan et al., 2017). Accordingly, both Dicer and the Argonaute protein eiF2c, a core component of the miRISC, are found in post-synaptic densities of dendritic spines (Lugli et al., 2005). Taken together, specific miRNAs have been established as key modulators of brain-specific signaling pathways associated with neuronal stem cell self-renewal, cell fate determination, neuronal and glial cell differentiation and proliferation, neurite growth, neurogenesis, synapse development and plasticity (Kosik, 2006; Schratt et al., 2006; Lugli et al., 2008; Fiore et al., 2009; Schratt, 2009; Siegel et al., 2009; Gao, 2010; Shi et al., 2010; Perruisseau-Carrier et al., 2011; de Chevigny et al., 2012; Wakabayashi et al., 2014; Stappert et al., 2015; Bielefeld et al., 2017; Chen et al., 2018; Zampa et al., 2018).

## The multimodal diversity of GABAergic interneurons

**FIGURE 2:**
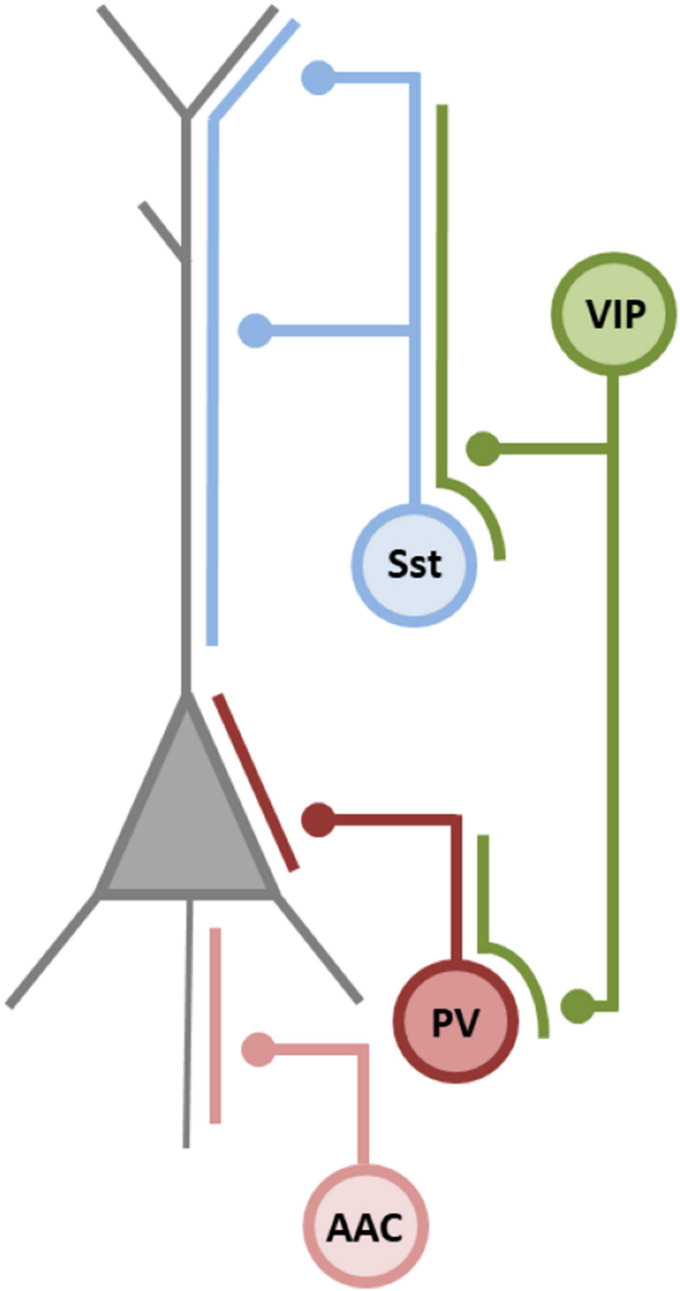
GABAergic interneuron overview. Cardinal classes of cortical IN differ in their morphology, neurochemical content, intrinsic electrophysiological properties and pattern of connectivity. For detailed description we refer to recent, excellent reviews on the classification and function of inhibitory INs (Tasic et al., 2016, 2018; Gouwens et al., 2019, 2020; Miyoshi, 2019). Parvalbumin (PV) INs synapse mainly on the somatic and perisomatic compartment of pyramidal cells, thereby controlling the spike generation in pyramidal cells. They are the major source of feedforward inhibition (Pouille and Scanziani, 2001; Freund, 2003; Mallet et al., 2005; Woodruff et al., 2009; Hu et al., 2014) and due to their divergent axonal targeting, they are able to synchronize large groups of postsynaptic neurons (Pouille and Scanziani, 2001; Ferguson and Gao, 2018; Yang and Sun, 2018; Missonnier et al., 2020). Consequently, they play pivotal roles in the generation and regulation of cortical rhythms, i.e., hippocampal theta rhythms, sharp wave ripples, and especially γ oscillations (Pouille and Scanziani, 2001; Sohal et al., 2009). Chandelier cells, or axo-axonic cells (AACs) are also PV expressing INs. They innervate the axon initial segment providing inhibition onto the spike initiation zone of pyramidal cells (Woodruff et al., 2009, but see Szabadics et al., 2006). Recently, it has been shown that AACs are active during heightened arousal and theta states (Dudok et al., 2021; Schneider-Mizell et al., 2021), thereby controlling CA1 pyramidal neurons outside of their place fields. Somatostatin (Sst) INs target the dendrites of pyramidal neurons (Kawaguchi and Kubota, 1996, 1997; Wang et al., 2004). Sst IN regulation of principal cell dendrites is critical for spine reorganization. Consequently, they play an important role in memory and learning processes (Chen et al., 2015; Honoré et al., 2021). Sst INs impact local circuits *via* feedback or lateral inhibition and have been shown to support cortical oscillations (Attinger et al., 2017; Muñoz et al., 2017; Obermayer et al., 2018). Vasoactive intestinal peptide (VIP) INs constitute the fourth major class of INs, comprising roughly 15% of all INs. VIP INs preferentially target other INs, mainly Sst, and, to a lesser degree, PV INs, thereby providing disinhibitory control over principal neurons (Rudy et al., 2011; Pi et al., 2013; Rhomberg et al., 2018). Thus, they constitute an important component of cortical disinhibitory circuits playing a role in gain control during sensory discrimination (Pi et al., 2013), and in cortical plasticity (Fu et al., 2015). Adapted from Fishell and Kepecs (2020).

Conventional classification uses various features to describe and categorize cortical INs (Ascoli et al., 2008). IN subtypes are placed in distinct subgroups according to morphological characteristics, intrinsic electrophysiological properties, as well as connectivity and protein expression patterns (Ascoli et al., 2008; Lee et al., 2010; Kepecs and Fishell, 2014; Tremblay et al., 2016; Tasic et al., 2018; Mihaljević et al., 2019). Recent developments in single cell transcriptomics added a new layer of complexity to IN classification (Tasic et al., 2016, 2018; Gouwens et al., 2019, 2020; Miyoshi, 2019). Gouwens et al. (2020) distinguished 28 types of cortical INs with congruent morphoelectrical and transcriptomic characteristics (so called met-types). Hierarchical clustering of IN properties revealed five major IN categories which were complementary, non-overlapping and designated by the expression of specific molecular markers: the calcium binding protein parvalbumin (PV), the neuropeptide somatostatin (Sst), the vasoactive intestinal peptide (VIP), the lysosomal-associated membrane protein family member 5 (LAMP5), and synuclein gamma (SNCG); the latter two subclasses mainly representing neurogliaform INs and cholecystokinin (CCK) INs, respectively. These categories overlap to a great extent with the cardinal IN subclasses distinguished according to their developmental and spatiotemporal origin in the medial or caudal ganglionic eminence (MGE or CGE, respectively), as described below (Fishell and Kepecs, 2020; Gouwens et al., 2020). Interestingly, the classification of met-types not only recapitulates the distinction of cardinal IN cell types based on developmental origin, but also reveals a layer-specific axon innervation pattern as a defining feature that distinguishes different met-types (Kawaguchi and Kubota, 1997; Klausberger and Somogyi, 2008; DeFelipe et al., 2013; Gouwens et al., 2020). In other words, the axonal projection pattern separates transcriptomic IN subtypes and in this manner implicates a functional differentiation according to their projection pattern. One consequence of this diversity in axonal arborisation is a functional compartmentalization of inhibition (Lovett-Barron et al., 2012; Royer et al., 2012; Fishell and Kepecs, 2020; Bloss et al., 2016). However, the implications of a granular differentiation among transcriptomic IN subtypes warrants further investigation, especially as recent observations indicate that, e.g., PV INs display a form of plasticity where they can adapt their molecular profile, intrinsic properties and connectivity pattern to changes in the local circuitry (Donato et al., 2013; Caroni, 2015; Dehorter et al., 2015). On a broader level, differentiation upon axonal projection patterns segregates IN into four major classes: (1) INs that project onto the soma of pyramidal neurons (PV INs), (2) INs that project onto the axon initial segment of pyramidal neurons (axo-axonic cells, or chandelier cells, also PV expressing), and (3) INs that project onto the dendrites of pyramidal neurons (Sst INs); finally, a fourth class consists of INs that project onto other INs (VIP INs). One interpretation of this diversity in axonal patterning is a division of labor of highly specialized inhibitory synapses (Huang et al., 2007; Klausberger and Somogyi, 2008; Fishell and Kepecs, 2020). For the remainder of this review, we will use this classification scheme as a guideline to relate miRNA-dependent control of gene expression in different IN classes to cortical inhibition. A short characteristic of major IN subclasses is presented in Figure 2.

## Differences in interneurons across species

There are major differences between rodents and primates in the proportion of glutamatergic principal cells and GABAergic INs, as well as among IN subtypes (Jones, 2009). The relation of pyramidal neurons vs. INs is approximately 2:1 in humans as compared to 5:1 in mice. Nearly 50% of GABA INs in rodents express PV, while approximately 20% are VIP immunoreactive. In primates, only around 20% of GABAergic INs are PV positive. Since IN subtypes integrate within cortical circuits in distinct manners, these dissimilarities are expected to differentially impact local and global network functioning. In contrast to rodents, in the fetal human forebrain two independent lineages of cortical INs have been distinguished (Letinic et al., 2002; Zecevic et al., 2011). While the subcortical ganglionic eminence (GE) is the primary source of rodent INs, the developmental origin of neocortical GABAergic INs in humans and non-human primates is still under debate. However, recent studies suggest that the majority of primate neocortical GABAergic INs may originate from GEs of the ventral telencephalon, similarly to rodents (Petanjek et al., 2009; Ma et al., 2013; Yu et al., 2021). Moreover, while the neurogenesis period varies within the GE subregions and across species, major IN classes and their migratory routes are evolutionarily ancient and remain well conserved (López-Bendito et al., 2008).

## Development of GABAergic cortical interneurons

Fate-mapping experiments revealed that cardinal IN types can be predicted based on their spatiotemporal origin, at the time when IN become postmitotic (Nery et al., 2002; Xu et al., 2003; Taniguchi et al., 2013; Mayer et al., 2018; Fishell and Kepecs, 2020). The precursors of cardinal GABAergic IN subgroups are primarily generated in the subpallidum in the ventral telencephalon (Wonders and Anderson, 2006; Batista-Brito and Fishell, 2009; Corbin and Butt, 2011). In rodents, neurogenesis and proliferation of GABAergic INs precursors occurs in the MGE and CGE and to a lesser extent in the preoptic area (POA), a subregion of the hypothalamus (Wonders and Anderson, 2006; Batista-Brito and Fishell, 2009; Gelman et al., 2009; Gelman and Marín, 2010; Corbin and Butt, 2011; Sultan et al., 2013). Each of these areas generates distinct IN subtypes depending on specific gene regulatory networks implemented by spatially and temporally restricted transcription factor activity (Kessaris et al., 2014). Collectively, MGE and CGE constitute the embryonic source of > 90% of GABAergic INs in the murine cerebral cortex (Wonders and Anderson, 2006; Batista-Brito and Fishell, 2009). MGE-derived INs are the major source of cardinal PV and Sst INs (Marín and Rubenstein, 2001; Xu et al., 2003; Butt et al., 2005; Wonders and Anderson, 2006; Fogarty et al., 2007; Miyoshi et al., 2010; Bandler et al., 2017) and their specification is mediated by numerous transcription factors including the Dlx family, the Nkx2 family, Lhx6, and Sox6 (Wonders and Anderson, 2006; Fogarty et al., 2007; Huang et al., 2007; Butt et al., 2008; Batista-Brito and Fishell, 2009). CGE-derived INs express the transcription factors Sp8, COUP-TF2, Prox1, and Pax6, resulting in IN subpopulations that closely overlap with the cardinal subgroup of VIP cells and other smaller cardinal subgroups (Pleasure et al., 2000; Xu et al., 2003; Butt et al., 2005; Lee et al., 2010; Miyoshi et al., 2015; Tremblay et al., 2016; Lim et al., 2018; Fishell and Kepecs, 2020). Upon their generation, postmitotic cortical INs migrate tangentially from the subpallium along the subventricular and marginal zone to the cortical plate, switch their migration pattern and travel radially into the developing cortical plate to finally reach their destination in the postnatal cortex (Faux et al., 2012; Wamsley and Fishell, 2017). Like IN generation and cardinal specification, migration and settling are complex processes regulated by an intricate network of various motogens, chemoattractants, transcription factors, and neurotransmitters (Marín and Rubenstein, 2001; De Marco García et al., 2011; Wamsley and Fishell, 2017; Lim et al., 2018). These developmental programs are regulated not only by intrinsic IN activity, but also by the forming immature neuronal circuits (Hurni et al., 2017; Bugeon et al., 2021). In addition, the early excitatory nature of GABA adds another layer of complexity to the multidimensional processes governing IN laminar positioning and integration within cortical circuits (Ben-Ari, 2007). Taken together, during migration and settling interaction of developing INs with extrinsic local cues promotes additional functional subtype diversity and finally shape IN morphology to establish their local connectivity pattern.

## miRNA significance for GABAergic interneuron development

Deep miRNA sequencing during cortical IN differentiation of human induced pluripotent stem cells (hiPSCs) revealed dynamic alterations of miRNA profiles across different stages of development (Tu et al., 2018). Specific miRNA expression patterns were observed at four time points: D0, D11, D25, D80, representing hiPSCs, neuron progenitor cells, immature neurons, and mature neurons, respectively. The generated miRNomes at D0 and D11 and those generated at D25 and D80 clustered together. While the miRNA-302 family, miRNA-372, and miRNA-367 were specifically highly expressed at the hiPSCs stage, the let-7 family, miRNA-9, and miRNA-124 were enriched in mature INs. Interestingly, the -3p and -5p forms were not always expressed consistently during neuronal differentiation, indicating that miRNA strand switching might affect developmental processes as well. Thus, dynamic changes of miRNA patterns reflect a complex regulatory mechanism governing distinct stages of neuronal differentiation as well as the emergence of final cortical IN cell types.

Tuncdemir et al. (2015) examined the impact of miRNA depletion (by means of Dicer knockout) in MGE-derived IN proliferation, migration, and differentiation by removing Dicer from MGE-progenitors as well as post-mitotic MGE-derived INs in mice. The loss of miRNAs impacted neither proliferation nor the initiation of migration. However, miRNAs were essential for the transition from tangential to radial migration and the subsequent survival and maturation of cortical INs, resulting in a profound reduction of cortical INs at postnatal day 21 (Tuncdemir et al., 2015). Furthermore, almost 50% of the fate-mapped neurons lost their cardinal signature (PV or Sst) and showed defects in their morphology. Interestingly, despite the reduction of INs at postnatal day 21, a precocious expression of Sst, neuropeptide Y (NPY) and glutamic acid decarboxylase 65 (GAD65) was observed in E15.5 Dicer mutant animals, indicative of a miRNA-dependent expression of specific IN markers. Finally, the transcription factors Lhx6, Sox6, and Satb1 were not changed in Dicer-mutant mice, arguing that miRNA-dependent mechanisms do not act through the previously demonstrated transcription factor networks in MGE-derived IN specification (Batista-Brito et al., 2009; Lee et al., 2010; Tremblay et al., 2016; Lim et al., 2018). Taken together, these results indicate that miRNA-dependent gene expression can regulate migration, maturation and specification of cortical INs, adding another regulatory layer to the previously described transcription factor programs.

The conditional removal of Dicer in postmitotic VIP INs in mice resulted in a progressive loss of VIP INs in adulthood, despite normal migration and maturation (Qiu et al., 2020). Before significant cell loss of VIP INs in superficial layers of the somatosensory and motor cortices, VIP INs displayed profound changes in intrinsic and synaptic properties. VIP INs had broader action potential (AP) half-width and smaller AP amplitudes. Furthermore, the frequency of miniature excitatory postsynaptic currents (mEPSCs) as well as miniature inhibitory postsynaptic currents (mIPSCs) was reduced. Concomitant to these changes, pyramidal neurons were affected as well: they displayed increased mIPSC frequencies and amplitudes as well as increased mEPSC frequencies. Surprisingly, behavioral testing revealed an improved spatial working memory and motor coordination performance (Qiu et al., 2020). In a follow-up study, Wu et al. (2022), characterized the effect of Dicer ablation in postmitotic VIP INs in the olfactory bulb. They observed disrupted odor processing and discrimination in mutant mice, as well as disturbed beta oscillations and theta coherence between the olfactory bulb (OB) and the anterior piriform cortex (Wu et al., 2022). Importantly, the Dicer ablation restricted to the olfactory bulb VIP INs recapitulated the behavioral and electrophysiological results of the global knockout (Wu et al., 2022).

Conditional deletion of Dgcr8, a part of the canonical microprocessor complex, in postmitotic cortical pyramidal neurons (Dgcr8^fl/fl^ mice, crossed to Nex-Cre mice) induced a profound reduction of their soma size and a loss of dendritic complexity in the cortex of mice resulting in an overall reduction of brain size in these animals (Hsu et al., 2012). These findings were recapitulated, when knocking out Dicer in Dicer^fl/fl^;Nex-Cre mice (Hong et al., 2013). However, in contrast to the deletion of Dicer, knocking out Dgcr8 was accompanied by a selective reduction of the PV IN population and perisomatic inhibitory synapses (Hsu et al., 2012). This non-cell autonomous effect was attributed to a disrupted brain-derived neurotrophic factor (BDNF)/tropomyosin receptor kinase B (TrkB) signaling pathway in Dgcr8^fl/fl^; Cre mice. Alternations in the number and function of PV IN population have been frequently observed in schizophrenia (Ferguson and Gao, 2018). More specifically, schizophrenia has been linked to deficits in the excitatory recruitment of PV INs in the ventral hippocampus and medial prefrontal cortex (mPFC) (Gonzalez-Burgos et al., 2015; Glausier and Lewis, 2018; Dienel and Lewis, 2019). Interestingly, Dgcr8 haploinsufficiency contributes to neurological, behavioral, and anatomical phenotypes of the 22q11 Deletion Syndrome (22q11DS), that encompasses DiGeorge syndrome, velo-cardio-facial syndrome and conotruncal anomaly face syndrome (Schofield et al., 2011). 0.6–2% of schizophrenia cases have been attributed to the 22q11DS microdeletion and approximately 30% of individuals with 22q11DS develop some type of schizophrenia in adolescence or adulthood (Green et al., 2009; Sellier et al., 2014). Consequently, 22q11DS has been proposed to represent a genetic subtype of schizophrenia (Bassett and Chow, 1999; Liu et al., 2002). Dgcr8 haploinsufficient mice (Dgcr8 ±) displayed reduced expression of miRNAs in the brain and showed cognitive deficits, along with altered electrical properties of layer 5 pyramidal neurons in the mPFC, decreased complexity of basal dendrites, and reduced excitatory synaptic transmission (Schofield et al., 2011). In an 22q11.2DS mouse model for schizophrenia (Lgdel ± mice), Mukherjee et al. (2019) observed a chronic PV plasticity state with reduced PV and glutamate decarboxylase 67 (GAD67) expression. In these mice, bidirectional PV plasticity and therefore the molecular, synaptic, and intrinsic adaptation of PV INs to changing levels of neural activity is disrupted, indicative of a maladjustment of PV INs to an excitatory recruitment deficit. Consequently, Lgdel ± mice displayed profound network and cognitive dysfunctions with reduced high-gamma oscillatory activity in the mPFC as well as behavioral deficits e.g., in con-specific and in object interaction. However, if and how Dgcr8 haploinsufficiency and consequently dysregulation in the post-transcriptional control of miRNA expression is implicated in the phenotypic changes in Lgdel ± mice, remains to be determined.

## miRNA “signature” for subtypes of GABAergic interneurons

Recent advances in genomic profiling have allowed to identify specific miRNA patterns across various cell types and tissues, some of which also displayed changes in expression patterns upon altered physiological states and in response to environmental cues (Weber et al., 2010; Kriegel et al., 2013; van Spronsen et al., 2013; Londin et al., 2015; Kuosmanen et al., 2017). To a considerable extent, the identity and activity of neuronal subpopulations can be determined by their gene expression profile, which in principle also includes miRNA expression patterns (Nelson et al., 2006; Hobert, 2008; He et al., 2012). In this manner, a systematic analysis of miRNA profiles in distinct IN subtypes would represent a first step toward establishing a link between cell phenotypes, miRNA expression and finally their contribution to neuronal circuit dynamics.

Using miRNA tagging and affinity-purification (miRAP) targeted to cell types through the Cre-loxP binary system, He et al. (2012) revealed distinct miRNA profiles in glutamatergic neurons and in subtypes of GABAergic INs in the neocortex and cerebellum of mice. miRNA profiles of neurons expressing GAD65, PV, and Sst clustered more closely together as compared to glutamatergic neurons. Moreover, they clustered together with Purkinje cells, a class of GABAergic inhibitory neurons in the cerebellum, implying that miRNA profiles are specific for neuron subtypes that share the neurotransmitter phenotype as well as a common developmental origin (He et al., 2012). When comparing PV and Sst subpopulations, 125 out of 511 detected miRNAs were differentially expressed. For example, miRNA-133b was significantly enriched in the PV cells, while miRNA-187 was more abundant in Sst cells (He et al., 2012). Along these lines, transcriptional profiling of PV immunoreactive neurons isolated postmortem from layer 3 of the superior temporal gyrus from schizophrenic patients revealed a differential expression for 15 miRNAs (hsa-miRNA-151-3p, hsa-miRNA-338-5p, hsa-miRNA-106a, hsa-miRNA-197, hsa-miRNA-342-3p, hsa-miRNA-518f, hsa-miRNA-1274b, hsa-miRNA-151-3p, hsa-miRNA-195, hsa-miRNA-197, hsa-miRNA-218, hsa-miRNA-342-3p, hsa-miRNA-34a, hsa-miRNA-361-5p, hsa-miRNA-520c-3p). The subsequent analysis of the predicted miRNA targets revealed elements of signaling pathways that overlap with those found to be unbalanced in schizophrenia (Pietersen et al., 2014).

Taken together, these data suggest that PV IN network disruptions may be at least partially mediated by gene network dysregulations due to altered expression of a rather small number of miRNAs (Pietersen et al., 2014) and that differentially expressed miRNAs might serve as a “signature” for GABAergic IN subtypes and regulate different subtype-specific functions.

## Olfactory bulb interneurons

The OB is regarded as an independent developmental domain (López-Mascaraque and de Castro, 2002) and provides an example where particular miRNAs have been shown to determine distinct developmental trajectories (Zolboot et al., 2021), rendering OB INs an attractive model to study cell type- and context-dependent miRNA regulation of signaling pathways. The mammalian OB contains two IN subpopulations of different spatiotemporal origin: INs generated during embryogenesis and the early postnatal period from local OB progenitor cells, and INs deriving from subventricular adult progenitors during the early postnatal period and adulthood (Vergaño-Vera et al., 2006; Alonso et al., 2012). These two subgroups present distinct morphological and physiological characteristics and are thought to play different roles in odor discrimination. Interestingly, miRNA-125, the mammalian homolog of lin-4 linked to regulation of neuronal differentiation and synaptic function (Sokol et al., 2008), is expressed only in OB INs from the subventricular zone. Sponging miRNA-125 resulted in enhanced dendritic morphogenesis and increased activation upon odor stimulation in adult born OB INs, indicative of an instructive role for miRNA-125 in the integration of adult born INs into OB circuitry (Akerblom et al., 2014).

In OB INs, not only developmental but also activity dependent regulation of gene expression is controlled by miRNAs. Sustained exposure of sibling larvae to kin odorants induces changes in neurotransmitter expression from GABA to dopamine (DA) in *Xenopus* accessory olfactory bulb (AOB) INs, accompanied by behavioral preference for kin odorants (Dulcis et al., 2017). Vice versa, prolonged exposure of sibling larvae to non-kin odorants drives a DA-to-GABA shift in AOB neurons paralleled by an aversion-to-attraction shift in social preference toward the same non-kin odorants. By means of small RNA sequencing and functional interrogation, miRNA-375 and miRNA-200b were identified as key regulators mediating changes in DA vs. GABA expression. miRNA-375 was shown to inhibit the transcription factor Pax6, a main determinant of the dopaminergic phenotype in AOB (Ninkovic et al., 2010), whereas inhibition of miRNA-200b increased both Pax6 and Bcl11b mRNA levels in the AOB resulting in a reduction of GABAergic neurons and an increase in the DA neuron population (Dulcis et al., 2017).

## miRNA regulation of GABAergic interneuron function in physiology and pathology

### miRNA-138-5p

miRNA-138-5p has been shown to be involved in dendritic spine morphogenesis in cultured hippocampal pyramidal neurons (Siegel et al., 2009). However, recently a pivotal role for miRNA-138-5p in the regulation of PV inhibitory synaptic transmission in the mouse hippocampus has been reported (Daswani et al., 2022). miRNA-138-5p inactivation specifically in INs by viral injection of sponge transcripts or by Cre- mediated expression of sponge transcripts restricted to PV INs resulted in an increased frequency of mIPSC in murine CA1 pyramidal neurons. Sponge transcripts sequester endogenous miRNA, thereby leading to miRNA inactivation and the de-repression of cognate target genes (Ebert and Sharp, 2010). At the behavioral level, miRNA-138-5p inactivation was accompanied by short-term memory deficits (Daswani et al., 2022). Moreover, genes found to be upregulated in the hippocampus of miRNA-138-5p sponge expressing mice significantly overlapped with genes that were also unbalanced in schizophrenic patients. Specifically, the receptor tyrosine kinase ErbB4 was upregulated upon miRNA-138-5p sponging and subsequently validated as a direct miRNA-138-5p target (Daswani et al., 2022). ErbB4 is predominantly expressed in PV INs (Vullhorst et al., 2009; Neddens and Buonanno, 2010; Skirzewski et al., 2018). Neuregulins and their receptor ErbB4 are critical for the assembly of PV IN circuitry including their migration, axon and dendrite development, and synapse formation (Mei and Nave, 2014), and have been identified as schizophrenia susceptibility genes (Bennett, 2009; Banerjee et al., 2010; Neddens et al., 2011; Joshi et al., 2014; Mei and Nave, 2014). ErbB4 has been found in the axons, as well as on the postsynaptic side of PV INs at afferent excitatory and inhibitory inputs (Fazzari et al., 2010). Recently, it has been demonstrated that ErbB4 plays an important role in the local translation of synaptic genes (Bernard et al., 2022) and that ErbB4 is instructive for the induction of bidirectional PV plasticity in the mPFC (Chen et al., 2022). Finally, alterations in neuregulin1 (NRG1)-ErbB4 signaling have been demonstrated to alter memory performance. However, depending on the model, ablating ErbB4 in PV INs of hippocampal CA1 either enhance (Tian et al., 2017) or impair (Robinson et al., 2022) spatial and working memory performance.

Taken together, these observations indicate that the regulation of PV INs by miR-138-5p and its downstream target ErbB4 is critically involved in the homeostasis of mature hippocampal PV IN microcircuits. Furthermore, disturbances of miRNA regulation in PV INs induces short-term memory deficits in mice reminiscent to cognitive impairments frequently observed in patients suffering from schizophrenia (Del Pino et al., 2013).

### miRNA-137

miRNA-137, a brain-enriched miRNA, has been shown to be involved in neurogenesis, dendritic morphogenesis and synaptic plasticity (Szulwach et al., 2010; Chen et al., 2012), and has been identified as a candidate gene for the etiology of schizophrenia, bipolar disorder, and autism spectrum disorders (Devanna and Vernes, 2014; Yin et al., 2014; Abdolmaleky et al., 2021). In the PFC and the blood of redox dysregulated mice [glutamate-cysteine ligase modifier subunit (Gclm)-KO mice], oxidative stress was associated with an elevated miRNA-137 level, a decrease in cytochrome c oxidase subunit 6A2 (COX6A2) and mitophagy markers, an accumulation of damaged mitochondria, and disturbed PV IN function (Khadimallah et al., 2022). In early psychosis patients, corresponding changes were detected, i.e., an increase in exosomal miRNA-137, a decrease in COX6A2 and mitophagy markers in the plasma and a concomitant reduction of γ oscillatory activity in the EEG (Khadimallah et al., 2022). Consequently, inhibition of miRNA-137 in the cortex of Gclm-KO mice reversed the alterations in PV network and the decrease in COX6A2, indicative for an involvement of the miRNA-137/COX6A2 pathway in cortical PV IN circuit impairments typically observed in schizophrenia.

### miRNA-181a-5p

Mild traumatic brain injury (mTBI) can result in a permanent impairment of learning and memory. Within the dentate gyrus (DG) of the hippocampus, the hilar subregion is particularly sensitive to mTBI and disruption of hilar IN inhibitory input has been linked to cognitive deficits following mTBI (Hicks et al., 1993). In a mouse model of mTBI, miRNA-181a-5p antagomir injected intracerebroventricularly prior to closed-skull cortical impact reduced neuronal miRNA-181a levels, restored deficits in novel object recognition and increased PV expression in hilar INs (Griffiths et al., 2019). Furthermore, these changes were associated with a decrease in the mTBI-related DG hyperactivity. PV is known to buffer calcium influx in PV INs (Schwaller et al., 2002) and thereby might be involved in calcium-mediated excitotoxicity. By reinstating PV expression, miRNA-181a-5p antagomir could alleviate the imbalance between excitation and inhibition in the DG due to mTBI (Griffiths et al., 2019). Interestingly, the level of miRNA-181a-5p was also increased in the hippocampus of post-status epileptic rats (Ren et al., 2016; Kong et al., 2020). Moreover, inhibition of miRNA-181a-5p *via* miRNA-181a antagomir led to seizure suppression and evoked a neuroprotective response *via* sirtuin 1 upregulation (Kong et al., 2020), and caspase-3 activation involved in neuronal apoptosis (Ren et al., 2016). However, the role of miRNA-181a-5p regulation of PV IN function and its contribution to the excitatory-inhibitory balance warrants further investigation.

### miRNA-24

The transcription factor Sox6 is crucial for subtype determination of MGE-derived postmitotic INs by suppression of PV IN specification while inducing specification of Sst INs (Batista-Brito et al., 2009; Kelsom and Lu, 2013; Hu et al., 2017). Gestational and lactational exposure to three endocrine disrupting chemicals (EDCs) in rats resulted in a sex-specific impairment of hippocampus-dependent behaviors and alternations in expression patterns of particular IN subtypes. Male, but not female offspring exposed to EDCs displayed learning and memory deficits accompanied by a decrease in miRNA-24 level, upregulation of mRNA for transcription factor Sox6, Sox11, Pou2f2/Oct2, Pou3f2/Brn2, and downregulation of mRNA for PV in the hippocampus (Lichtensteiger et al., 2021). Individual Sox6 mRNA levels correlated inversely with miRNA-24 and PV mRNA expression. Moreover, mRNAs for NRG1 and its receptor ErbB4 were upregulated upon exposure to EDCs in male hippocampal INs, indicating that sex differences add an additional layer of post-transcriptional control of gene expression by miRNAs in PV INs.

### miRNA-218

The early postnatal period is a crucial time window regarding ultimate morphological differentiation and the proper integration of cortical INs within local networks. Recently, miRNA-218 has been demonstrated to regulate multiple aspects of neural circuit development in the early postnatal period (Taylor et al., 2022). Transient inhibition of miRNA-218 in the dorsal hippocampus in early postnatal life resulted in the disruption of early depolarizing GABAergic signaling, structural defects in dendritic spines in CA1, and increased intrinsic membrane excitability in CA3 pyramidal neurons resulting in a heightened hippocampal network activity and a predisposition to seizures. Previous work has shown that miRNA-218 is implicated in embryonic motor neuron development (Amin et al., 2015, 2021; Thiebes et al., 2015; Reichenstein et al., 2019), in homeostatic plasticity (Rocchi et al., 2019), in stress related responses (Torres-Berrío et al., 2020; Schell et al., 2022; Yoshino et al., 2022), as well as in regulating contextual and spatial memory processes (Lu et al., 2021). Surprisingly, transcriptional profiling revealed that the upregulated genes upon miRNA-218 inhibition were more enriched in INs as compared to pyramidal neurons (Taylor et al., 2022). Consequently, conditional knockout of miRNA-218 in INs, but not pyramidal neurons, was sufficient to recapitulate the effects on hippocampal network assembly. Taken together, these results suggest that miRNA-218 regulates IN function in early postnatal life, thereby coordinating hippocampal network assembly to establish proper E/I balance in the adult.

### miRNA-134

miRNA-134, one of the best-studied miRNAs in the brain, is highly activity-dependent and has been shown to regulate dendrite growth and dendritic spine formation in rat hippocampal pyramidal neurons (Schratt et al., 2006; Fiore et al., 2009; Bicker et al., 2014; Bahlakeh et al., 2021). Although its function in excitatory neurons has been well documented, using a ratiometric miRNA sensor Chai et al. (2013) surprisingly detected an activity-dependent upregulation of miRNA-134 in cortical INs that were immunoreactive for Sst or calretinin (CR), but not in pyramidal neurons. In Sst INs, miRNA-134 interacted directly with the mRNA encoding the palmitoylation enzyme DHHC9, which in turn regulated the proper membrane targeting of H-Ras. H-Ras has been implicated in multiple forms of plasticity in the developing visual cortex (Arendt et al., 2004; Kaneko et al., 2010). However, how H-Ras regulates Sst IN function is currently not known and warrants further investigation.

### Other miRNAs

Low GABAergic tone is increasingly implicated in the etiology of stress-related disorders (Ma et al., 2016; Zhang et al., 2017; Fogaça and Duman, 2019; Ma et al., 2019; Perlman et al., 2021). In the cortex of mice that underwent chronic unpredictable mild stress (CUMS), upregulation of several miRNAs was observed (miRNA-15b-5p, miRNA-144-3p, miRNA-582-5p and miRNA-879-5p). Stressed mice displayed impairments in GABA synthesis, reuptake, and release, indicative of an impairment in GABAergic signaling. Transcriptional profiling revealed a downregulation of GAD67, vesicular GABA transporter (VGAT) and GABA transporter type 3 (GAT-3) mRNAs which were subsequently shown to be negatively regulated by the upregulated miRNAs (Ma et al., 2016). Recent evidence from human postmortem and animal studies suggests a relatively selective vulnerability of Sst INs in depressive disorder, while changes in other INs seem to be less pronounced (Tripp et al., 2011; Lin and Sibille, 2015; Fee et al., 2017). However, to characterize the role of individual miRNAs and their target mRNAs in this specific IN subtype further investigation is required.

## Conclusion and future directions

The significance of post-transcriptional regulation of gene expression by miRNAs in the central nervous system (CNS) is mirrored by a growing number of studies linking dysregulation of miRNA pathways to various neurodevelopmental and neuropsychiatric disorders. While an important role for miRNAs in regulating the development and function of GABAergic INs is beginning to emerge, it is apparent that a more detailed characterization of individual miRNAs and their target mRNAs in specific IN types is needed. This line of research has the potential not only to increase our fundamental knowledge of the consequences of miRNA regulation of GABAergic INs, but also the mechanistic understanding of neuropsychiatric disorders with recognized GABAergic dysfunctions like schizophrenia, autism spectrum and affective disorders.

Genetic programs underlying IN development are orchestrated by both transcriptional and post-transcriptional regulation. Data presented in this review indicate that the phenotypic and physiological features of IN subtypes depend not only on developmental spatiotemporal patterning of transcription factor activity and environmental cues, but also on miRNA expression and function (Strobl-Mazzulla et al., 2012; Tuncdemir et al., 2015; Dulcis et al., 2017). Furthermore, the interactions between gene expression, inductive events and miRNA activity not only determine IN developmental pathways but also impact mature network organization (Lichtensteiger et al., 2021; Taylor et al., 2022). In this manner, miRNAs are important elements of the gene regulatory network contributing to IN specification (Strobl-Mazzulla et al., 2012; Dulcis et al., 2017), as well as to the modification of network assemblies during critical developmental periods (Lichtensteiger et al., 2021; Taylor et al., 2022).

In addition, INs display remarkable plasticity features in an experience-dependent and behaviorally specific manner. They can adapt their molecular profile, their intrinsic and synaptic properties to changing levels of neuronal activity (Donato et al., 2013; Dehorter et al., 2015, 2017). However, there is a gap of knowledge in linking gene expression programs of INs to circuit modification mechanistically. miRNA-dependent post-transcriptional regulation of gene expression might be a prominent candidate to fill this gap as miRNA-dependent regulation of central aspects of principal neuron development and plasticity has been demonstrated (Schratt et al., 2006; Siegel et al., 2009; McNeill and Van Vactor, 2012; Aksoy-Aksel et al., 2014). Daswani et al. (2022) observed developmentally independent modifications in PV IN microcircuitry due to miRNA-138-5p inhibition in a cell type-specific manner. However, if these changes are plastic, i.e., if they are modified bidirectionally and in an activity-dependent manner, remains to be determined. Despite these first observations, the precise contribution of miRNAs to PV IN plasticity and to possible plasticity features of other IN subtypes remains elusive.

An essential step toward understanding the regulatory role of miRNAs in GABAergic INs is an extensive portrayal of miRNome profiles in a cell type-specific manner, in the relevant developmental trajectories as well as in mature microcircuitry. Obviously, this poses major technical challenges, particularly due to the high heterogeneity of GABAergic INs. Recent progress in sequencing technologies has provided a first step toward the analysis of differential miRNA expression, thus allowing to discriminate between neurons and glia cells (Colin et al., 2009), brain regions (Bak et al., 2008; Minami et al., 2014) as well as cell types (He et al., 2012). However, single-cell small RNA sequencing techniques and consequently a finer granularity of analysis are only beginning to emerge (Smith and Hutvagner, 2022). Moreover, a fine-grain analysis of the role of specific miRNAs in a cell type-specific manner is complicated by the pleotropic ability of single miRNAs to regulate multiple biological pathways. Therefore, a more comprehensive characterization of the miRNome-targetome interactions is required (Keaveney et al., 2020). The recognition of the biological relevance of a particular miRNA and its targeted molecular pathways will foreseeably be facilitated by advances in bioinformatics, transcriptomics, proteomics and other “omics” approaches. Together with elaborate molecular tools such as antagomirs, “sponges”, miRNA mimics and precursors, as well as cell type-specific Cre-driver transgenic mouse lines that are intended to silence or overexpress miRNAs, the path to reveal distinct miRNA-dependent biological processes in a cell type-specific manner is set (Issler and Chen, 2015). Finally, a comprehensive knowledge of the role of miRNAs in GABAergic INs may be instrumental in elucidating the molecular basis of many CNS diseases with recognized GABAergic dysfunction. As neuronal miRNAs are responsive to environmental changes and are actively secreted by cells, they may additionally constitute useful diagnostic and prognostic biomarkers for the respective disease (van den Berg et al., 2020; Tsermpini et al., 2022).

## Author contributions

JW and KK: conception and design. KK: literature search. KK, GS, and JW: wrote the manuscript. All authors contributed to the article and approved the submitted version.
